# Supplementary material for: Tracking control of robotic manipulator end-effector trajectory based on robust sliding mode method
Source: PLoS One. 2025 Apr 15;20(4):e0320118. doi: 10.1371/journal.pone.0320118 (PMC11999709; doi:10.1371/journal.pone.0320118)
Supplement: S1 — (PDF) [file pone.0320118.s001.pdf]

%%%%%%%%%%%%%%%%%%%%%%%%%%%The main program of this paper is divided into several important modules: control input, controller and drawing.

Input model

```
%%%%%%%%%%%

function [sys,x0,str,ts] = spacemodel(t,x,u,flag)

switch flag,

case 0,

    [sys,x0,str,ts]=mdlInitializeSizes;

case 1,

    sys=mdlDerivatives(t,x,u);

case 3,

    sys=mdlOutputs(t,x,u);

case {2,4,9}

    sys=[];

otherwise

    error(['Unhandled flag = ',num2str(flag)]);

end

function [sys,x0,str,ts]=mdlInitializeSizes

sizes = simsizes;

sizes.NumContStates  = 0;

sizes.NumDiscStates  = 0;

sizes.NumOutputs     = 6;

sizes.NumInputs      = 0;

sizes.DirFeedthrough = 1;

sizes.NumSampleTimes = 1;

sys = simsizes(sizes);

x0  = [];
```

```
str = [];
ts    = [0 0];
function sys=mdlOutputs(t,x,u)
xd1=cos(t);
d_xd1=-sin(t);
dd_xd1=-cos(t);
xd2=sin(t);
d_xd2=cos(t);
dd_xd2=-sin(t);
sys(1)=xd1;
sys(2)=d_xd1;
sys(3)=dd_xd1;
sys(4)=xd2;
sys(5)=d_xd2;
sys(6)=dd_xd2;

%%%%%%%%%%%%%%%%%%%%%% main control
function [sys,x0,str,ts]=s_function(t,x,u,flag)
switch flag,
case 0,
    [sys,x0,str,ts]=mdlInitializeSizes;
case 1,
    sys=mdlDerivatives(t,x,u);
case 3,
    sys=mdlOutputs(t,x,u);
case {2, 4, 9 }
    sys = [];
```

```
otherwise
        error(['Unhandled flag = ',num2str(flag)]);
end
function [sys,x0,str,ts]=mdlInitializeSizes
global J Fx
sizes = simsizes;
sizes.NumContStates    = 4;
sizes.NumDiscStates    = 0;
sizes.NumOutputs         = 8;
sizes.NumInputs           = 6;
sizes.DirFeedthrough = 1;
sizes.NumSampleTimes = 0;
sys=simsizes(sizes);
x0=[1 0 1 0];
str=[];
ts=[];
J=0;Dx=0;Cx=0;Gx=0;Fx=[0 0];
function sys=mdlDerivatives(t,x,u)
global J Fx
xd1=u(1);
d_xd1=u(2);
dd_xd1=u(3);
xd2=u(4);
d_xd2=u(5);
dd_xd2=u(6);

l1=1;l2=1;
```

```
P=[1.46 0.402 0.75 3.025 1.095];
g=9.8;
L=[l1^2 l2^2 l1*l2 l1 l2];

pl=0.485;

M=P+pl*L;
Q=(x(1)^2+x(3)^2-l1^2-l2^2)/(2*l1*l2);
q2=acos(Q);
dq2=-1/sqrt(1-Q^2);

A=x(3)/x(1);
p1=atan(A);
d_p1=1/(1+A^2);

B=sqrt(x(1)^2+x(3)^2+l1^2-l2^2)/(2*l1*sqrt(x(1)^2+x(3)^2));
p2=acos(B);
d_p2=-1/sqrt(1-B^2);

if q2>0
    q1=p1-p2;
    dq1=d_p1-d_p2;
else
    q1=p1+p2;
    dq1=d_p1+d_p2;
end
J=[-sin(q1)-sin(q1+q2) -sin(q1+q2);
```

```
        cos(q1)+cos(q1+q2) cos(q1+q2)];
d_J=[-dq1*cos(q1)-(dq1+dq2)*cos(q1+q2) -(dq1+dq2)*cos(q1+q2);
        -dq1*sin(q1)-(dq1+dq2)*sin(q1+q2) -(dq1+dq2)*sin(q1+q2)];

D=[M(1)+M(2)+2*M(3)*cos(q2) M(2)+M(3)*cos(q2);
        M(2)+M(3)*cos(q2) M(2)];
C=[-M(3)*dq2*sin(q2) -M(3)*(dq1+dq2)*sin(q2);
        M(3)*dq1*sin(q2)    0];
G=[M(4)*g*cos(q1)+M(5)*g*cos(q1+q2);
      M(5)*g*cos(q1+q2)];

Dx=(inv(J))'*D*inv(J);
Cx=(inv(J))'*(C-D*inv(J)*d_J)*inv(J);
Gx=(inv(J))'*G;

e1=xd1-x(1);
e2=xd2-x(3);
de1=d_xd1-x(2);
de2=d_xd2-x(4);
e=[e1;e2];
de=[de1;de2];

Hur=15*eye(2);
r=de+Hur*e;

dxd=[d_xd1;d_xd2];
dxr=dxd+Hur*e;
```

```
ddxd=[dd_xd1;dd_xd2];
ddxr=ddxd+Hur*de;

K=30*eye(2);
Fx=Dx*ddxr+Cx*dxr+Gx+K*r;

dx=[x(2);x(4)];
S=inv(Dx)*(Fx-Cx*dx-Gx);

sys(1)=x(2);
sys(2)=S(1);
sys(3)=x(4);
sys(4)=S(2);
function sys=mdlOutputs(t,x,u)
global J Fx

tol=J'*Fx;

sys(1)=x(1);
sys(2)=x(2);
sys(3)=x(3);
sys(4)=x(4);
sys(5:6)=Fx(1:2);
sys(7:8)=tol(1:2);

%%%%%%%%%%%%%%%%%%%%%%%%%%%%%%%%%main    plot
close all;
```

```
figure(1);
subplot(211);
plot(t,xd(:,1),'r',t,x(:,1),'b--','linewidth',3);
xlabel('time(s)');ylabel('position tracking of x axis');
legend('Ideal x','Actual x');
subplot(212);
plot(t,xd(:,1)-x(:,1),'k','linewidth',3);
xlabel('time(s)');ylabel('Tracking error of x axis');
legend('Error of x axis');

figure(2);
subplot(211);
plot(t,xd(:,4),'r',t,x(:,3),'b--','linewidth',3);
xlabel('time(s)');ylabel('position tracking of y axis');
legend('Ideal y','Actual y');
subplot(212);
plot(t,xd(:,4)-x(:,3),'k','linewidth',3);
xlabel('time(s)');ylabel('Tracking error of y axis');
legend('Error of y axis');

figure(3);
subplot(211);
plot(t,xd(:,2),'r',t,x(:,2),'b--','linewidth',3);
xlabel('time(s)');ylabel('Velocity tracking of x axis');
```

```
legend('Ideal dx','Actual dx');
subplot(212);
plot(t,xd(:,2)-x(:,2),'m','linewidth',3);
xlabel('time(s)');ylabel('Error of x-axis velocity tracking');
legend('Error of dx');


figure(4);
subplot(211);
plot(t,xd(:,5),'r',t,x(:,4),'b--','linewidth',3);
xlabel('time(s)');ylabel('velocity tracking of y axis');
legend('Ideal dy','Actual dy');
subplot(212);
plot(t,xd(:,5)-x(:,4),'m','linewidth',3);
xlabel('time(s)');ylabel('Error of y-axis velocity tracking');
legend('Error of dy');

figure(5);
plot(t,x(:,5),'r',t,x(:,6),'b--','linewidth',3);
xlabel('time(s)');ylabel('Conrol input Fx1 and Fx2');
legend('Fx of first link','Fx of second link');

figure(6);
```

```
plot(t,x(:,7),'r',t,x(:,8),'b--','linewidth',3);

xlabel('time(s)');ylabel('Conrol input tol1 and tol2');

legend('tol of first link','tol of second link');




figure(7);

plot(xd(:,1),xd(:,4),'r','linewidth',3);

hold on;

plot(x(:,1),x(:,3),'b--','linewidth',2);

xlabel('x');ylabel('y');

legend('Ideal trajectory','Practical    trajectory');
```
