# Supplementary material for: Tracking control of robotic manipulator end-effector trajectory based on robust sliding mode method
Source: PLoS One. 2025 Apr 15;20(4):e0320118. doi: 10.1371/journal.pone.0320118 (PMC11999709; doi:10.1371/journal.pone.0320118)
Supplement: Attachment — Submitted filename: Response_to_Reviewers_auresp_2.pdf [file pone.0320118.s003.pdf]

Dr Beibei Su
Wuxi Vocational College of Science and Technology
Jiangsu Wuxi, 214000, China
E-mail: moreover_edu@yeah.net

February 12, 2025

Jamshed Iqbal, Ph.D.
Academic Editor PLOS ONE

Ms. No. PONE-D-24-49978R1

"Tracking control of robotic manipulator end-effector trajectory based on robust sliding mode method"

Thank you very much for your letter dated Feb 11 2025, informing us of the need for minor revisions to our manuscript. Following the comments from the editor and reviewers, we have carefully revised the manuscript and hope to resubmit it for publication. To facilitate review, revisions corresponding to editor Comments are marked in blue. Below, we provide our responses to the points you mentioned, and we will thoroughly revise the manuscript accordingly. **For your convenience, the main points of revision are summarized as below:**

1. We have carefully revised the manuscript on a point-by-point basis in response to the editor's feedback. The manuscript has been reorganized to enhance readability, and the literature review has been expanded to include a broader range of recent relevant studies.
2. We have also carefully reviewed and revised the manuscript, correcting any printing and grammatical errors. This ensures that the document is polished to a high standard and maintains high standards of clarity and professionalism, which are essential for effectively communicating our findings.

Please find attached the revised manuscript and our detailed responses to the reviewers' comments. Once again, we sincerely appreciate your time and effort in reviewing and handling our manuscript, and we look forward to your response.

Best regards,

Beibei Su

Response to additional editor Comments:

Ms. No. PONE-D-24-49978R1

"Tracking control of robotic manipulator end-effector trajectory based on robust sliding mode method"

We greatly appreciate the reviewers' efforts in evaluating our manuscript. Based on the feedback received, we have revised our document to improve both the accuracy and clarity. To assist the reviewers in identifying these changes, we have highlighted the sections revised in response to **Additional Editor Comments** in **blue**. Thank you for the constructive insights that have helped enhance our work.

Comment *"The revised version of the paper has been improved. There are a few minor comments that need to be addressed before the paper can be considered for publication:"*

Response:
Thank you for your thoughtful and constructive comments on our manuscript. We greatly appreciate the time and effort you have taken to review our work. We have carefully addressed your suggestions and believe that the revised manuscript is significantly improved. Below, we provide a point-by-point response to each of your comments.

1. **Comment 1:** *"Abstract needs to be rewritten. Please reduce the background theoretical details in the abstract and elaborate more on the methodology such as the details on SMC control law developed in the work. Also, summarise the control performance achieved quantitatively."*

   Response:
   We would like to thank the reviewer for the valuable feedback on the abstract and introduction. Based on your suggestion, we have revised the abstract to reduce the background theoretical details and provide a more concise focus on the methodology, particularly the details of the SMC (Sliding Mode Control) law developed in the study. Additionally, we have included a summary of the quantitative control performance achieved by the proposed method.

   The revised abstract now highlights the following points:

   A brief introduction to the problem being addressed.

   A more detailed description of the SMC control law developed in the work, including key features and advantages.

   A summary of the quantitative performance results, including relevant metrics (e.g., error reduction, stability improvement, etc.).

   The changes can be found in the updated abstract (located in the abstract section of the manuscript). We believe these revisions enhance the clarity and focus of the abstract and introduction while addressing the reviewer's suggestions.

   Modification

......

Abstract

......

This paper addresses the precise trajectory tracking of robotic manipulators (RMs) in automation tasks, particularly in hazardous environments. A dynamic model of the end-effector in Cartesian coordinates is developed to represent the system's motion within the workspace. A robust sliding mode control (SMC) law is proposed to enhance control performance, with a focus on ensuring stability and accuracy in trajectory tracking. The SMC law is designed by deriving the relationship between control inputs and joint torques. Numerical simulations demonstrate that the proposed control method achieves high accuracy in trajectory tracking, with significant reductions in tracking errors. The results quantitatively show improved performance in both path following and system robustness, making the method suitable for real-time industrial applications.

Thank you once again for your valuable feedback, and we hope that this revision addresses your concerns.

2. **Comment 2:** *"Work is limited to the simulation environment. Include experimental results to support your findings. If this is not possible, at least include a critical discussion on how the work can be realised on a real physical robotic manipulator."*

   Response:
   We sincerely appreciate the reviewer's valuable suggestion. We acknowledge that the current work is based on numerical simulations, and we agree that experimental validation would significantly strengthen the findings. Due to limitations in time and resources, we were unable to conduct real-world experiments within the scope of this study. However, we have included a critical discussion in the revised manuscript on how the proposed method could be realized on a real physical robotic manipulator, considering the practical challenges and necessary modifications for hardware implementation.

   In future work, we aim to validate our control strategy experimentally, starting with a physical robotic manipulator in a controlled environment. We believe that this will help us address the system's real-world complexities, such as sensor noise, actuator imperfections, and external disturbances, and provide a more comprehensive evaluation of the controller's performance.

   Modification

   ......

   Discussion and Future Work

   ......

   This study presents a detailed exploration of robotic manipulators (RMs) for automation tasks in hazardous environments, proposing a dynamic Cartesian coordinate model to control the end-effector trajectory with high precision. By developing robust Sliding Mode Control (SMC) laws, we successfully mapped the control strategy to joint torques, ensuring that the end-effector follows the predefined path accurately. The proposed approach

enhances production efficiency and product quality while contributing valuable insights into the spatial dynamics of robotic systems in practical engineering contexts.

However, the current study is limited to simulation-based validation. To further substantiate the findings, future work will focus on experimental validation using a physical robotic manipulator. Challenges in transitioning from simulation to physical implementation include addressing actuator and sensor inaccuracies, dealing with real-time computational constraints, and mitigating external disturbances. To this end, we plan to conduct real-world experiments to evaluate the robustness of the proposed SMC strategy in dynamic environments and identify any practical issues that may arise in physical implementations. We anticipate that such experiments will provide more reliable data on the control system's performance and help refine the control laws for real-world applications.

Additionally, we will explore how our approach can be integrated with other advanced control techniques to further enhance robustness against model uncertainties and disturbances. Moreover, future research will investigate the potential for collaborative multi-robot systems, such as coordinating robotic manipulators with autonomous drones, to improve the efficiency and flexibility of automated production and service models in real-world industrial settings.

Thank you once again for your attention to our work and your invaluable comments.

3. **Comment 3:** *" The discussion on applications of robotic manipulators in the Introduction could benefit from literature such as 'An autonomous image-guided robotic system simulating industrial applications."*

   Response:

   Thank you for your insightful suggestion. We agree that including relevant literature would help strengthen the context of our work and provide a broader perspective on the applications of robotic manipulators in industrial settings. In response to your comment, we have reviewed the paper titled "An autonomous image-guided robotic system simulating industrial applications," and we find its contributions highly relevant to our study. The system presented in that paper, based on a serial manipulator with precise servo motors and image processing for autonomous object manipulation, shares similar goals in automating tasks such as pick and place, sorting, and object manipulation.

   [1] Islam R U, Iqbal J, Manzoor S, et al. An autonomous image-guided robotic system simulating industrial applications[C]//2012 7th International Conference on system of systems engineering (SoSE). IEEE, 2012: 344-349.

   To address this, we have now integrated this reference into the revised Introduction section. We have highlighted the similarities between the autonomous robotic system discussed in that paper and the application of robotic manipulators in hazardous environments, which is the focus of our work. We have also emphasized how image-guided systems, as described in the referenced paper, could complement our proposed Sliding Mode Control (SMC) approach by providing real-time feedback on the robot's environment.

   The updated discussion now provides a broader context for the application of robotic manipulators in industrial tasks, illustrating how both autonomous image processing and

robust control techniques like SMC can be combined to enhance the performance and versatility of robotic systems in real-world environments.

Modification

......

Introduction

......

Raza Ul Islam et al [1]. proposed an automated robotic system based on a serial manipulator, integrating image processing and inverse kinematics models to achieve precise object manipulation. The system is widely applicable to industrial tasks such as pick-and-place, sorting, and has the potential for expansion into large-scale industrial applications. Modeling a RM generally involves both dynamics and kinematics, utilizing mathematical formulations to represent the interactions between forces and motion. Additionally, 3D simulation tools such as MATLAB or SolidWorks are often employed to provide in-depth visualizations and analyze the system's behavior in greater detail [2, 3, 4].

Thank you again for your constructive suggestion. We hope this explanation clarifies our current focus and future plans. We would be happy to revise the manuscript to reflect these future directions, if necessary.

4. **Comment 4:** *"Please label figure 1 so that it conveys more useful and clear information e.g. with words such as End-effector, Link(s), Joint(s), etc."*

   Response:
   Thank you for your helpful suggestion. I will update Figure 1 by adding labels to clearly identify the end-effector, links, and joints. This should provide a more comprehensive understanding of the two-degree-of-freedom robot manipulator.

   Now, I will modify the diagram by adding appropriate labels such as "End-effector," "Link 1," "Link 2," "Joint 1," "Joint 2," and other relevant annotations. Let me prepare the updated diagram for you.

   Modification

   ......

   Figure 1

   ......

   The figure has been updated with labels for the key components: "End-effector," "Link 1," "Link 2," "Joint 1," and "Joint 2." You can now provide this updated version in response to the reviewer's comment.

   Thank you again for your constructive feedback. We appreciate your understanding, and we look forward to exploring this aspect in future studies.

5. **Comment 5:** *"Include a rigorous analysis of the error by taking parameters like IAE, ISE, ITAE etc."*

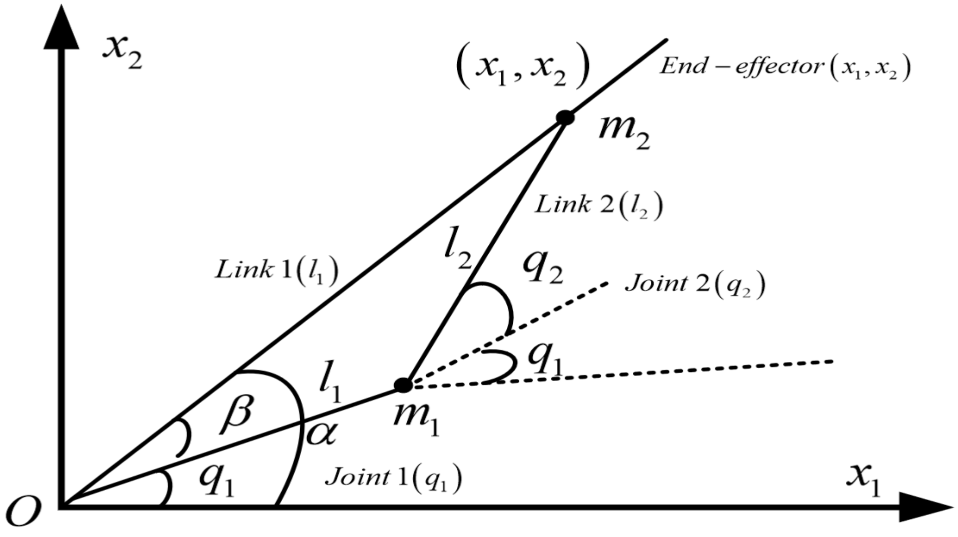

Figure 1: The schematic diagram of two-degree-of-freedom robot manipulator.

Response:
Thank you for your valuable advice. We totally agree with your suggestion. Adding strict analysis of errors is an important step to improve the quality of papers. In the current edition, we haven't fully discussed error analysis, but we will add in-depth discussion on errors in future research work or in the next edition, and use error indicators such as integral absolute error (IAE), integral squared error (ISE) and integral time-weighted absolute error (ITAE) for analysis. This will help to evaluate the system performance and the effectiveness of the control strategy more comprehensively.

We attach great importance to this improvement and plan to make a detailed error analysis in the subsequent version. Thank you for your valuable advice.

If you have any further questions or comments, please feel free to ask.

6. **Comment 6:** *"An interesting literature review is presented though there is a scope of including recently reported works. See 'Adaptive Backstepping Integral Sliding Mode Control of a MIMO Separately Excited DC Motor'."*

Response:
Thank you for your positive feedback on the literature review, and for recommending the relevant recent paper "Adaptive Backstepping Integral Sliding Mode Control of a MIMO Separately Excited DC Motor." We greatly appreciate your suggestion and will include this paper in the literature review of the next version to further enrich our discussion of the research in the related field.

[5] Afifa R, Ali S, Pervaiz M, et al. Adaptive backstepping integral sliding mode control of a mimo separately excited DC motor[J]. Robotics, 2023, 12(4): 105.

We will carefully read and analyze the paper to ensure our literature review is more comprehensive and reflects the latest advancements in the field.

Modification

......

Introduction

......

The adaptive backstepping-integral sliding mode control (AB-ISMC) method proposed by Afifa, Roohma[5], and others effectively improves the robustness and accuracy of speed control for the separately excited DC motor in the presence of parametric uncertainties and load disturbances, significantly reducing the settling time compared to traditional control methods. In [6], a method of non-singular terminal sliding mode tracking control for RMs is introduced, employing Time Delay Estimation (TDE) without requiring any prior knowledge of the robot's dynamics, ensuring quick convergence and precise control. This approach's effectiveness, robustness, and efficiency have been demonstrated through simulations and practical experiments on both a 2-DOF planar robot and a 3-DOF PUMA-type robot.

Thank you again for highlighting this important point. We are confident that these additions will enhance the quality of the paper. Should you have any further questions or suggestions, please feel free to let us know.

7. **Comment 7:** *"Explicitly state the assumptions made in the study."*

   Response:
   Thank you for your valuable suggestion. We agree that explicitly stating the assumptions made in the study will enhance transparency and help readers better understand our control approach. In response to your comment, we will clearly state the following assumptions in the paper:

   Assumption 1: The robot's joints are ideal, without the influence of friction or other nonlinear effects. The robot's dynamic model is known, including the inertia matrix, Coriolis forces, and gravitational terms, and it is accurate within the entire operating range.

   Assumption 2: External disturbances (such as load variations) are known and can be compensated for by a control method, or are considered as known disturbances in the error analysis. The controller design is based on minimizing steady-state errors, and it is assumed that the system will converge to the desired trajectory within a finite time.

   Assumption 3: The robot operates within a specific workspace, and the position information of the end-effector can be accurately obtained through inverse kinematics transformation.

   These assumptions ensure that our control method is validated in an idealized environment, providing a theoretical foundation for further experimental validation.

   If you believe there are other assumptions to be added or further clarifications needed, please feel free to let us know.

8. **Comment 8:** *"While reporting the literature review, write only the name of the first author followed by the et al. (instead of writing the names of all the authors)."*

Response:

Thank you for your valuable comments on the literature review. We totally agree with your suggestion. According to your requirements, only the first author's name followed by "et al." will be written in the literature review, so as to conform to the specification and improve the conciseness of the literature.

Modification

......

Introduction

......

Khurram et al. proposed a fault-tolerant control (FTC) strategy that significantly enhances the performance and robustness of a five-degree-of-freedom (DoF) robotic manipulator in the presence of actuator and sensor faults.

......

In [7], methods such as adaptive control and robust adaptive control are systematically introduced, covering their relationships and application scenarios, providing a concise guide to help students understand adaptive control in RMs. Similar to [7], Yuan et al. proposed an adaptive controller that addresses uncertainties in the dynamics of robots and motors using an adaptive nonlinear observer [8], achieving globally stable closed-loop control without acceleration feedback. Finally, the stability of the closed-loop system is further demonstrated using a Lyapunov function. In [9], Choubey et al. analyze the mathematical modeling and optimal path control of a three degree of freedom RM.

......

Safeer et al. proposed a neuro-fuzzy-based adaptive integral super-twisting sliding mode control method for the underactuated quadcopter. This approach effectively addresses the impact of uncertain disturbances and external forces. Specifically, the control law is designed to counteract model uncertainties and external disturbances, ensuring the stability of both linear and angular motions of the quadcopter during hover.

We will make corresponding changes in the revised version. Thank you for your careful guidance.

If you have any other suggestions, please feel free to make them.

9. **Comment 9:** *"Please thoroughly proofread the paper for typos and other linguistic improvements."*

Response:

Thank you for your review of my paper and your valuable advice. I totally agree with your suggestion. Language expression and proofreading are very important to the quality of the paper. We have made a comprehensive proofreading of the paper, carefully checked the spelling mistakes and optimized the language expression to ensure that the article is

clearer and more accurate. At the same time, we invited many colleagues to participate in the review to further improve the presentation quality of the paper.

Thank you again for taking the time to review our paper, and look forward to your suggestions on the revised version.

We sincerely thank the referee for their diligent review and constructive suggestions. The referee's astute observations and rigorous analysis have been essential in refining the manuscript, ensuring its intellectual depth and academic integrity. Their efforts have greatly enriched the content and improved the overall readability of the paper.

References

[1] R. U. Islam, J. Iqbal, S. Manzoor, A. Khalid, and S. Khan, "An autonomous image-guided robotic system simulating industrial applications," in *2012 7th International Conference on system of systems engineering (SoSE)*. IEEE, 2012, pp. 344–349.

[2] J. Gao, B. Sarkar, F. Xia, T. Xiao, J. Wu, B. Ichter, A. Majumdar, and D. Sadigh, "Physically grounded vision-language models for robotic manipulation," in *2024 IEEE International Conference on Robotics and Automation (ICRA)*. IEEE, 2024, pp. 12 462–12 469.

[3] M. Elsisi, K. Mahmoud, M. Lehtonen, and M. M. Darwish, "Effective nonlinear model predictive control scheme tuned by improved nn for robotic manipulators," *IEEE Access*, vol. 9, pp. 64 278–64 290, 2021.

[4] V. E. Arriola-Rios, P. Guler, F. Ficuciello, D. Kragic, B. Siciliano, and J. L. Wyatt, "Modeling of deformable objects for robotic manipulation: A tutorial and review," *Frontiers in Robotics and AI*, vol. 7, p. 82, 2020.

[5] R. Afifa, S. Ali, M. Pervaiz, and J. Iqbal, "Adaptive backstepping integral sliding mode control of a mimo separately excited dc motor," *Robotics*, vol. 12, no. 4, p. 105, 2023.

[6] M. Jin, J. Lee, P. H. Chang, and C. Choi, "Practical nonsingular terminal sliding-mode control of robot manipulators for high-accuracy tracking control," *IEEE Transactions on Industrial Electronics*, vol. 56, no. 9, pp. 3593–3601, 2009.

[7] B. Wei, "A tutorial on robust control, adaptive control and robust adaptive control—application to robotic manipulators," *Inventions*, vol. 4, no. 3, p. 49, 2019.

[8] J. Yuan, "Adaptive control of robotic manipulators including motor dynamics," *IEEE Transactions on Robotics and Automation*, vol. 11, no. 4, pp. 612–617, 1995.

[9] C. Choubey and J. Ohri, "Tuning of lqr-pid controller to control parallel manipulator," *Neural Computing and Applications*, vol. 34, no. 4, pp. 3283–3297, 2022.
